# Compositional changes in fecal microbiota in a new Parkinson's disease model: C57BL/6-Tg(NSE-haSyn) mice

**DOI:** 10.1186/s42826-023-00181-4

**Published:** 2023-11-15

**Authors:** Ji Eun Kim, Ki Chun Kwon, You Jeong Jin, Ayun Seol, Hee Jin Song, Yu Jeong Roh, Tae Ryeol Kim, Eun Seo Park, Gi Ho Park, Ji Won Park, Young Suk Jung, Joon Yong Cho, Dae Youn Hwang

**Affiliations:** 1 Department of Biomaterials Science (BK21 FOUR Program), College of Natural Resources and Life Science/Life and Industry Convergence Research Institute/Laboratory Animal Resources Center, Pusan National University, Miryang, Korea; 2 Exercise Biochemistry Laboratory, Korea National Sport University, Seoul, South Korea; 3 College of Pharmacy, Pusan National University, Busan, Korea

**Keywords:** Parkinson's disease, α-Synuclein, Gut–brain axis, Microbiota, Transgenic mice

## Abstract

**Background:**

The gut–brain axis (GBA) in Parkinson's disease (PD) has only been investigated in limited mice models despite dysbiosis of the gut microbiota being considered one of the major treatment targets for neurodegenerative disease. Therefore, this study examined the compositional changes of fecal microbiota in novel transgenic (Tg) mice overexpressing human α-synuclein (hαSyn) proteins under the neuron-specific enolase (NSE) to analyze the potential as GBA model.

**Results:**

The expression level of the αSyn proteins was significantly higher in the substantia nigra and striatum of NSE-hαSyn Tg mice than the Non-Tg mice, while those of tyrosine hydroxylase (TH) were decreased in the same group. In addition, a decrease of 72.7% in the fall times and a 3.8-fold increase in the fall number was detected in NSE-hαSyn Tg mice. The villus thickness and crypt length on the histological structure of the gastrointestinal (GI) tract decreased in NSE-hαSyn Tg mice. Furthermore, the NSE-hαSyn Tg mice exhibited a significant increase in 11 genera, including Scatolibacter, Clostridium, Feifania, Lachnoclostridium, and Acetatifactor population, and a decrease in only two genera in Ligilactobacillus and Sangeribacter population during enhancement of microbiota richness and diversity.

**Conclusions:**

The motor coordination and balance dysfunction of NSE-hαSyn Tg mice may be associated with compositional changes in gut microbiota. In addition, these mice have potential as a GBA model.

**Supplementary Information:**

The online version contains supplementary material available at 10.1186/s42826-023-00181-4.

## Background

The gut–brain axis (GBA) is the bidirectional neurohumoral communication between the central nervous system (CNS) in the brain and the enteric nervous system (ENS) in the gastrointestinal tract through immune, endocrine, humoral, and neural connections [1]. This communication includes CNS, neuroendocrine, neuroimmune, hypothalamic-pituitary-adrenal axis (HPA axis), autonomic nervous, ENS, vagus nerve, and gut microbiota [2, 3]. In particular, the brain can affect various intestinal effector cells via neural and hormonal communication, including epithelial cells, enteric neurons, smooth muscle cells, immune cells, interstitial cells of Cajal (ICC), and enterochromaffin cells [4]. These cells are affected by the gut microbiota through the regulation of mucus and biofilm production, gastrointestinal (GI) motility, GI permeability, and immune response [5]. Furthermore, the gut microbiota helps regulate the production of neurotransmitters, the protection of the intestinal barrier, enteric sensory afferents, bacterial metabolites, and mucosal immune function in the brain [1, 6]. The dysfunction of GBA was detected widely in several neurodegenerative diseases, such as Parkinson’s disease (PD), Alzheimer’s disease (AD), and Huntington's disease [7–9]. Therefore, GBA has attracted considerable attention as one of the treatment targets for these diseases.

The correlation between alteration in the gut microbiota composition and PD has been investigated in only a few models, even though most of them with PD phenotypes are rodents. thymocyte differentiation antigen 1 (Thy1) -alpha-synuclein (a-Syn) transgenic (Tg) mice exhibit dysbiosis of gut microbiota including *Proteus sp.*, *Bilophila sp.*, *Lachnospiraceae*, and *Verrucomicrobiae* during significant impairment in beam traversal, pole descent, and hindlimb reflex [10, 11]. A similar alteration in the gut bacterial composition was detected in the methyl-4-phenyl-1,2,3,6-tetrahydropyridine (MPTP)-induced mode with the PD phenotypes. The relative abundance of several microbial families, including *Lachnospiraceae*, *Prevotellaceae*, and *Erysipelotrichaceae*, increased or decreased remarkably in the PD model compared to the control group [12]. In addition, the bidirectional communication via the gut–brain axis was detected in Dendritic cell factor 1 (Dcf1) knockout (KO) mice. Three phyla, including *Bacteroidetes*, *Firmicutes*, and *Proteobacteria*, show significant changes in the microbiota composition between the wild-type (WT) and Dcf1 KO mice [13]. On the other hand, C57BL/6-Tg (NSE-hαSyn) mice which overexpressed hαSyn proteins under the control of the neuron-specific enolase (NSE) provided from National Institute of Food and Drug Safety Evaluation (NIFDS) in Korea as one of new PD model. This model showed activation on the DJ-1 mediated cytoprotective mechanism against oxidative stress as well as alteration on the lipoprotein profile [14]. But, C57BL/6-Tg (NSE-hαSyn) mice have never been used to study the correlation between the GBA and phenotypes of PD.

This study examined whether dysfunction of the motor neurons in NSE-hαSyn Tg mice can link to the dysbiosis of gut microbiota to verify the potential as a novel model for GBA.

## Results

### Verification of αSyn protein overexpression in the brain of NSE-hαSyn Tg mice

**Fig. 1:**
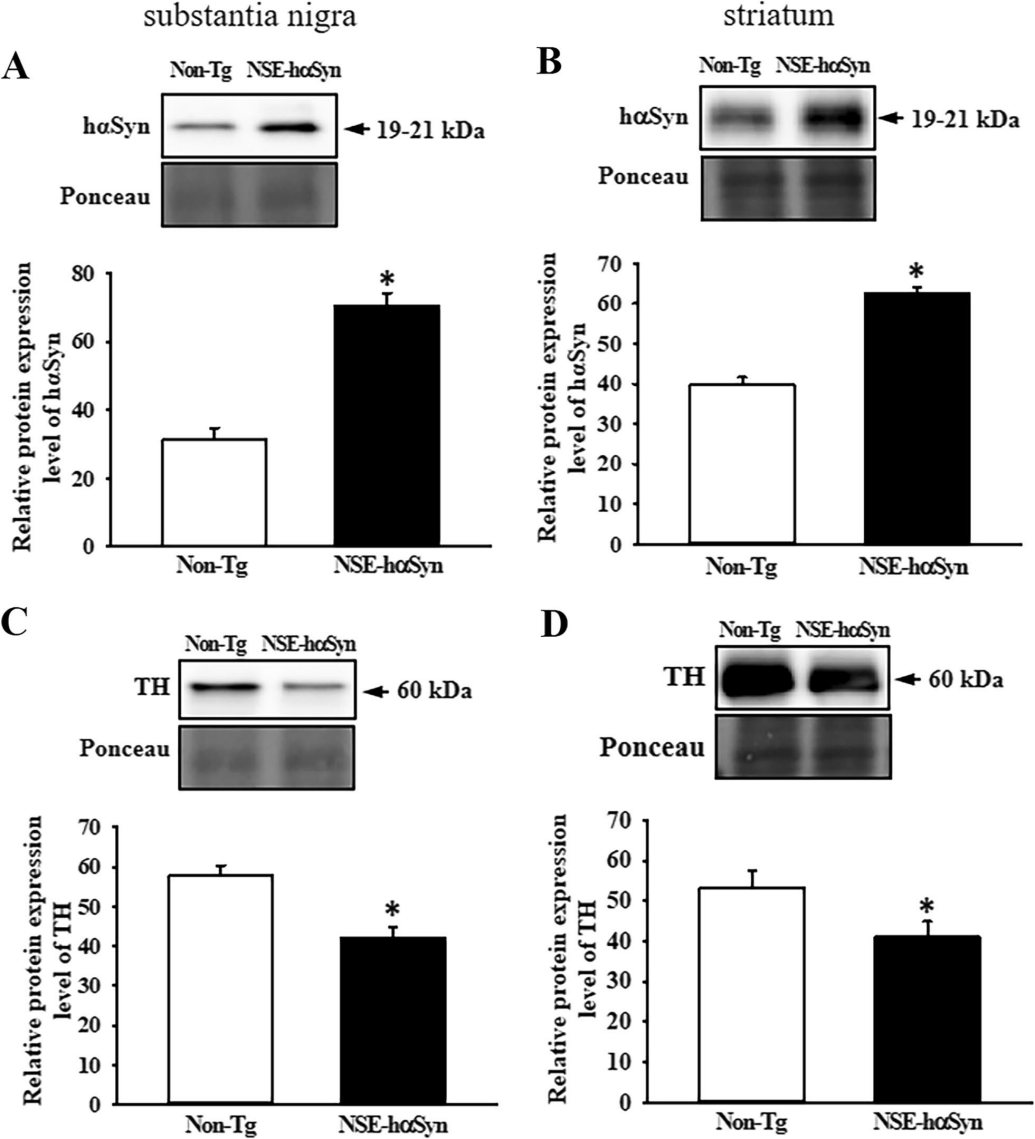
Expression levesl of hαSyn (**A** and **B**) and TH (**C** and **D**) proteins in the substantia nigra (**A** and **C**) and striatum (**B** and **D**) of NSE-hαSyn Tg mice. After detecting the haSyn and TH proteins using a specific antibody, the band density for this protein was analyzed using densitometry. The tissue homogenates were prepared on two to three tissues per group, and western blotting was analyzed twice for each sample. The level of each protein was normalized to β‑actin. The data were presented as the mean ± SD. *P < 0.05 versus Non-Tg group. Abbreviation: *hαSyn* human α synuclein, *TH* tyrosine hydroxylase, *NSE* neuron-specific enolase, *Tg* Transgenic mice

The levels of αSyn protein expression in the substantia nigra and striatum of NSE-hαSyn Tg mice were measured to verify the overexpression of αSyn protein under the control of the NSE promoter. A similar expression pattern was observed in the substantia nigra and striatum of NSE-hαSyn Tg mice. The level of this protein was significantly higher in NSE-hαSyn Tg mice than Non-Tg mice (p = 0.031 in substantia nigra, p = 0.001 in striatum) (Fig. 1A, B). Therefore, these results suggest that NSE-hαSyn Tg mice can serve as a model for PD by successfully expressing the causative gene.

### Reduction of TH expression in the brain of NSE-hαSyn Tg mice

**Fig. 2:**
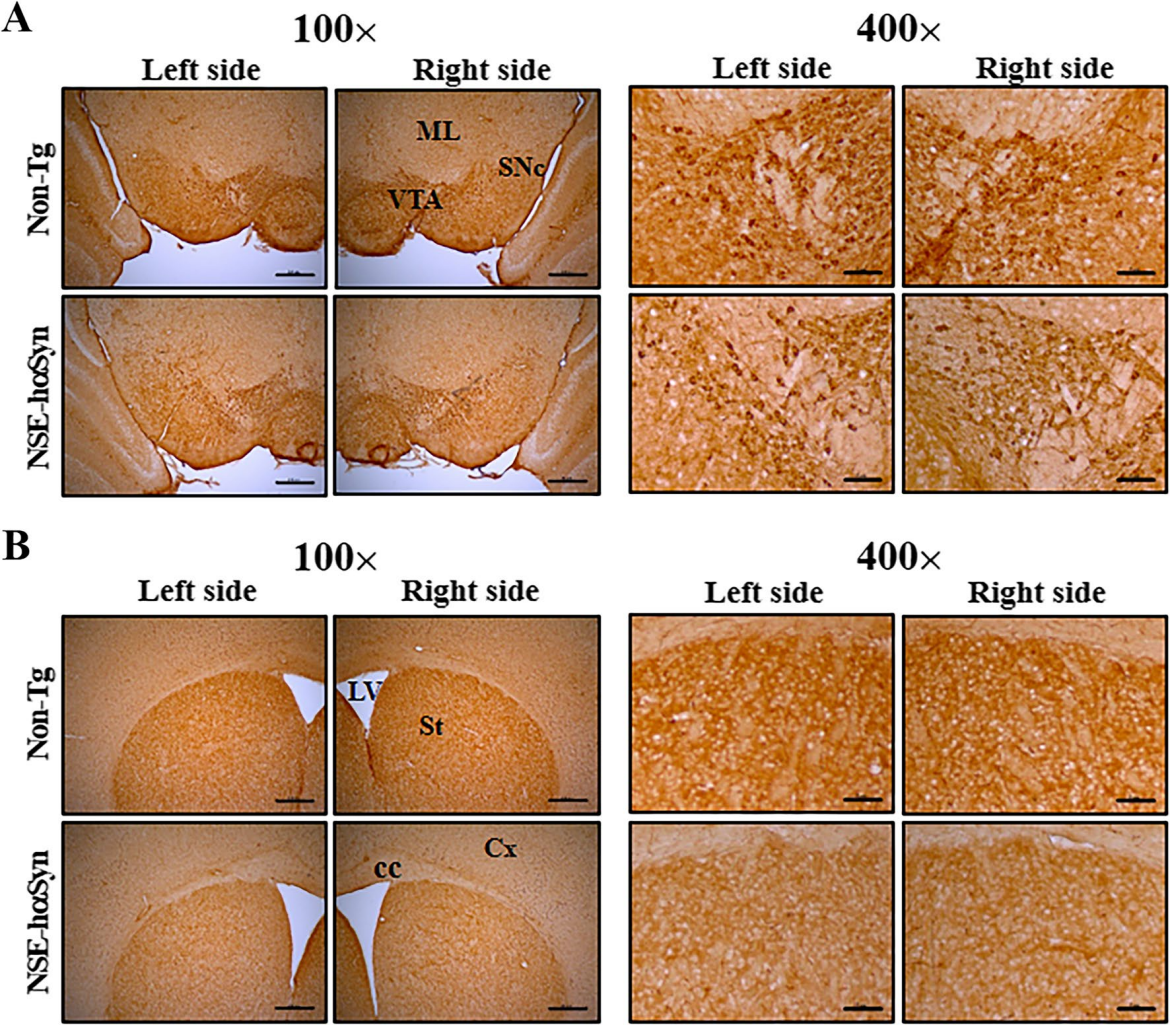
Tissue distribution of TH in the substantia nigra (**A**) and striatum (**B**) of NSE-hαSyn Tg mice. After staining specific antibodies, the stained brain section was observed by optical microscopy at 100x and 400x magnification. Brown or dark brown indicates the expression of TH in the cytoplasm of the substantia nigra and striatum. The immunostained slides were prepared from three to five mice per group, and the color density for each sample were observed in duplicate. Abbreviations: Abbreviation; *hαSyn* human α synuclein, *TH* tyrosine hydroxylase, *NSE* neuron-specific enolase, *Tg* transgenic mice, *ML* medial lemniscus, *VTA* ventral tegmental area, *SNc* substantia nigra pars compacta, *LV* lateral ventricle, *St* striatum, *Cx* cortex, *cc* corpus callosum

This study examined whether overexpression of αSyn protein is accompanied by changes in TH protein expression because a decrease in TH expression led to PD by diminishing dopamine synthesis [15]. The levels of TH proteins were measured in the substantia nigra and striatum of NSE-hαSyn Tg mice. This level was decreased significantly in both brain regions, even though the total expression level was higher in the striatum (p = 0.0001) than substantia nigra (p = 0.003) (Fig. 1C, D). In addition, these changes in the level of TH protein expression were reflected in the tissue distribution of the brain. The brown color density for TH proteins was lower in the substantia nigra and striatum of NSE-hαSyn Tg mice than in the Non-Tg mice (Fig. 2). These results show that overexpression of the αSyn protein may tightly associate with the reduction of TH expression in the brain of NSE-hαSyn Tg mice.

### Dysfunction of motor behavior performance in NSE-hαSyn Tg mice

**Fig. 3:**
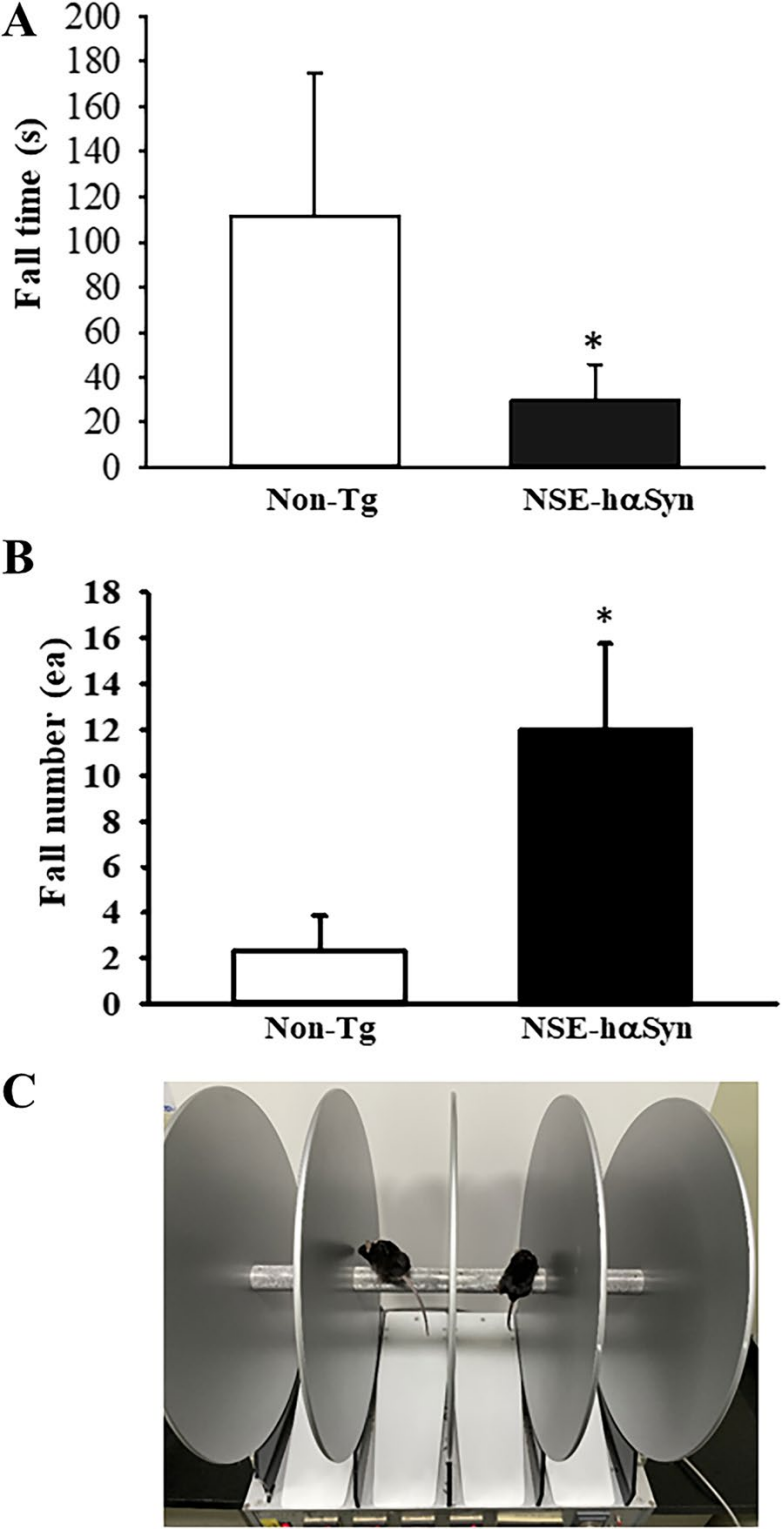
Rotarod test of NSE-hαSyn Tg mice. **A** Time to fall off the rod in the RotaRod test. **B** Number to fall off the rod in the RotaRod test. **C** Picture of mice on the rotarod. The time and number of falls were measured on five to six mice per mouse. The data are presented as the mean ± SD. *P < 0.05 versus non-Tg group. Abbreviation: *hαSyn* human α synuclein, *NSE* neuron-specific enolase, *Tg* Transgenic mice

The abnormalities in the motor behavior performance were analyzed in NSE-hαSyn Tg mice using the rotarod test to determine if a decrease in TH expression was accompanied by a dysfunction of motor coordination and balance. After 5 min on a rotating rod, the fall times decreased by 72.7% in NSE-hαSyn Tg mice compared to 90-week-old Non-Tg mice (p = 0.01), while fall number increased by 3.8 times in the same group (p = 0.002) (Fig. 3). Therefore, these results suggest that NSE-hαSyn Tg mice may exhibit the dysfunction of motor coordination and balance as a marker for PD.

### Alteration in the histopathological structure of the GI tract in the brain of NSE-hαSyn Tg mice

**Fig. 4:**
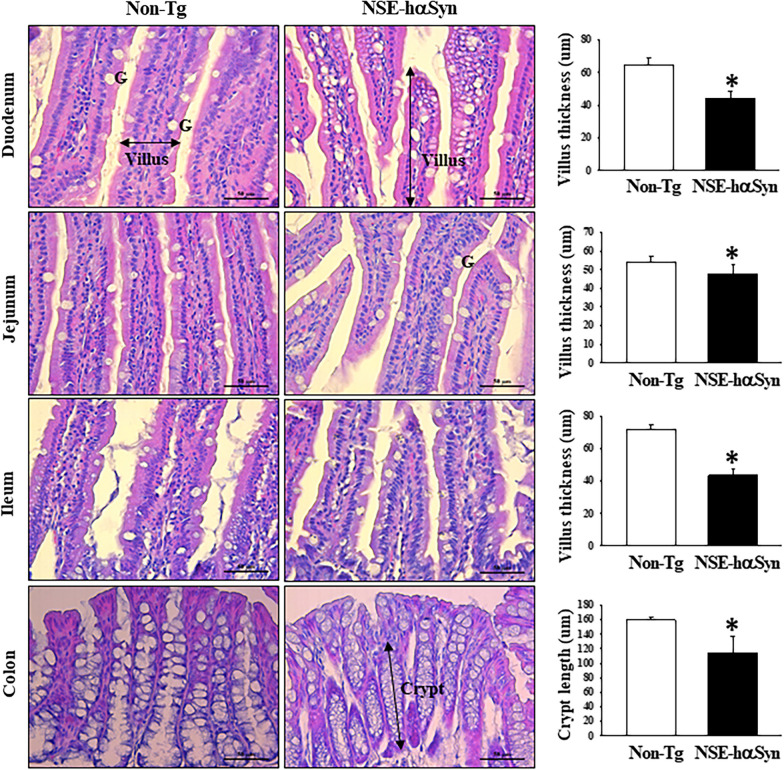
Histopathological structures in the GI tract of NSE-hαSyn Tg mice. H&E-stained sections of the GI tract were observed at 200x magnification using an optical microscope. The degree of histopathological changes in the retina tissue was measured using an Image J program. The H&E-stained slides were prepared from three to five mice per group, the histopathological parameter analyses were analyzed twice for each sample. The data was presented as the mean ± SD. *P < 0.05 versus Non-Tg group. Abbreviation; *GI* Gastrointestinal tract, *hαSyn* human α synuclein, *NSE* neuron-specific enolase, *Tg* Transgenic mice, *H&E* Hematoxylin and eosin

Next, this study examined whether the dysfunction of motor coordination and balance in NSE-hαSyn Tg mice was accompanied by alteration in the histopathological structure of the GI tract as the first evidence of the GBA. The histopathological structure of the GI tract was maintained constantly between Non-Tg and NSE-hαSyn Tg mice (Fig. 4A). On the other hand, the thickness of the villus was decreased significantly on the duodenum (p = 0.002), jejunum (p = 0.015), and ileum (p = 0.006) in NSE-hαSyn Tg mice compared to Non-Tg mice, while the crypt length was remarkably decreased in the same group (p = 0.002) (Fig. 4B). These results suggest that the dysfunction of motor coordination and balance in NSE-hαSyn Tg mice may be associated with changes in the histopathological structure of the GI tract.

### Alteration in the profile of the fecal microbiota of NSE-hαSyn Tg mice

**Fig. 5:**
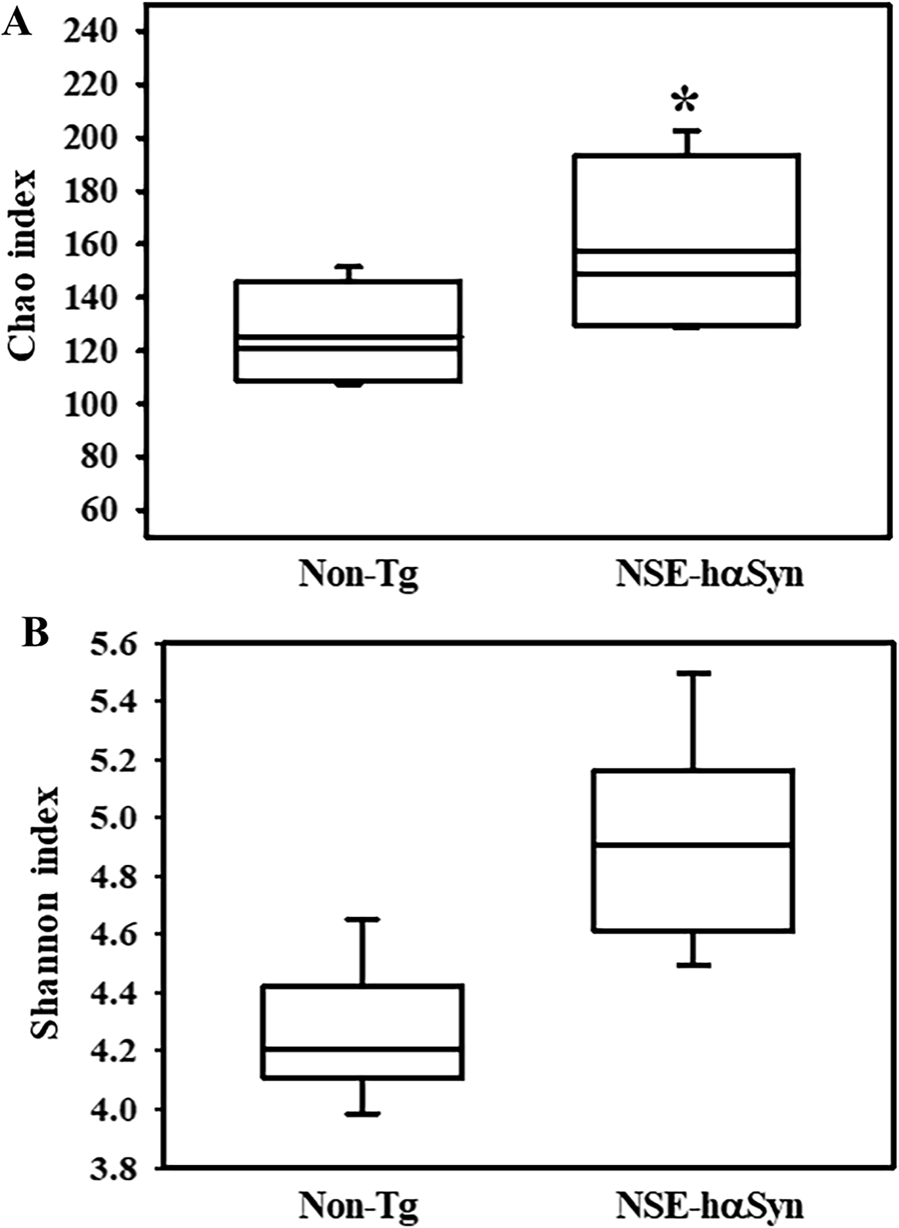
Microbial diversity analysis of NSE-hαSyn Tg mice. **A** Chao1 index. **B** Shannon index. The data are presented as the mean ± SD. *P < 0.05 versus Non-Tg group. Abbreviation: *hαSyn* human α synuclein, *NSE* neuron-specific enolase, *Tg* transgenic mice

**Fig. 6:**
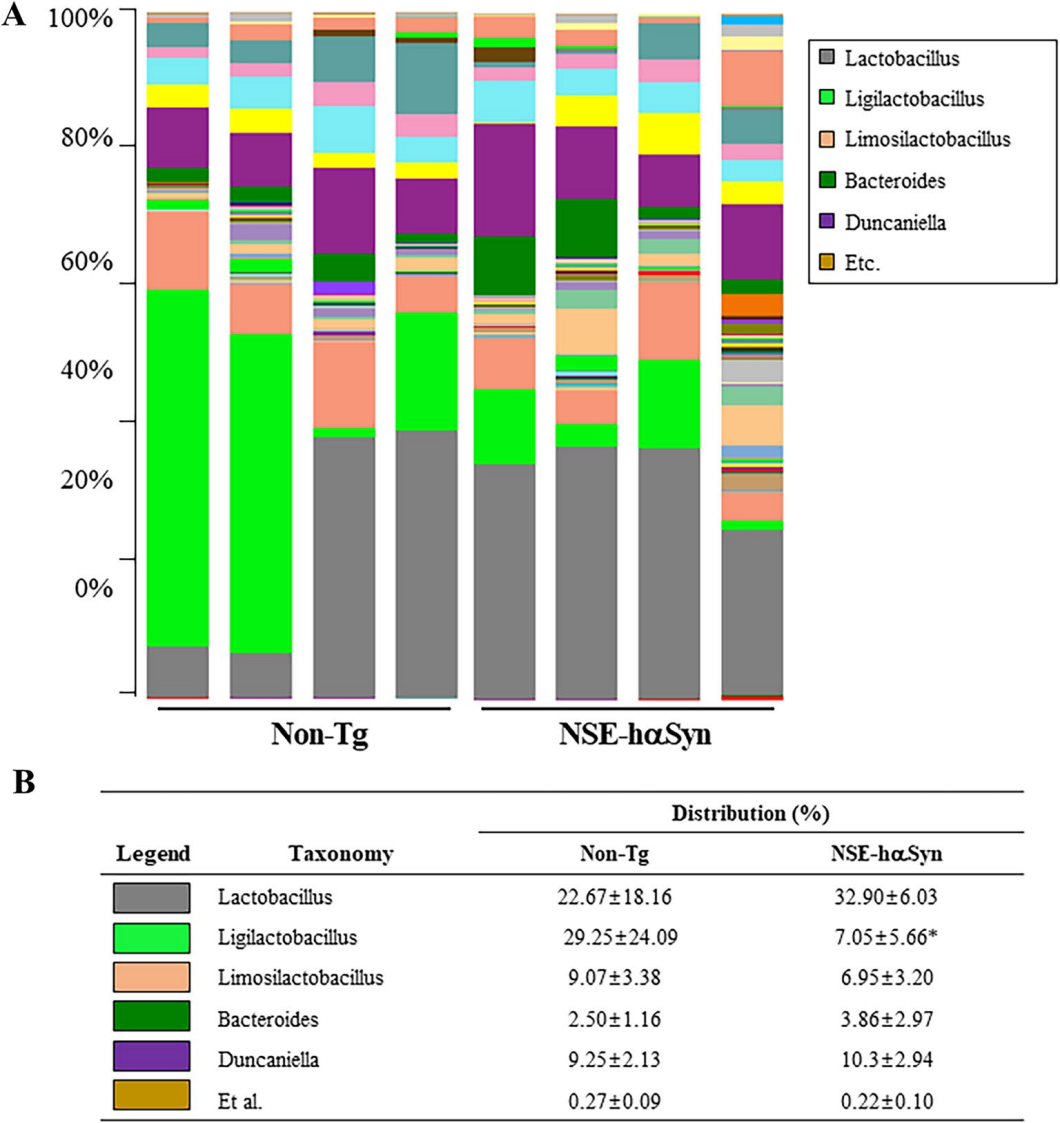

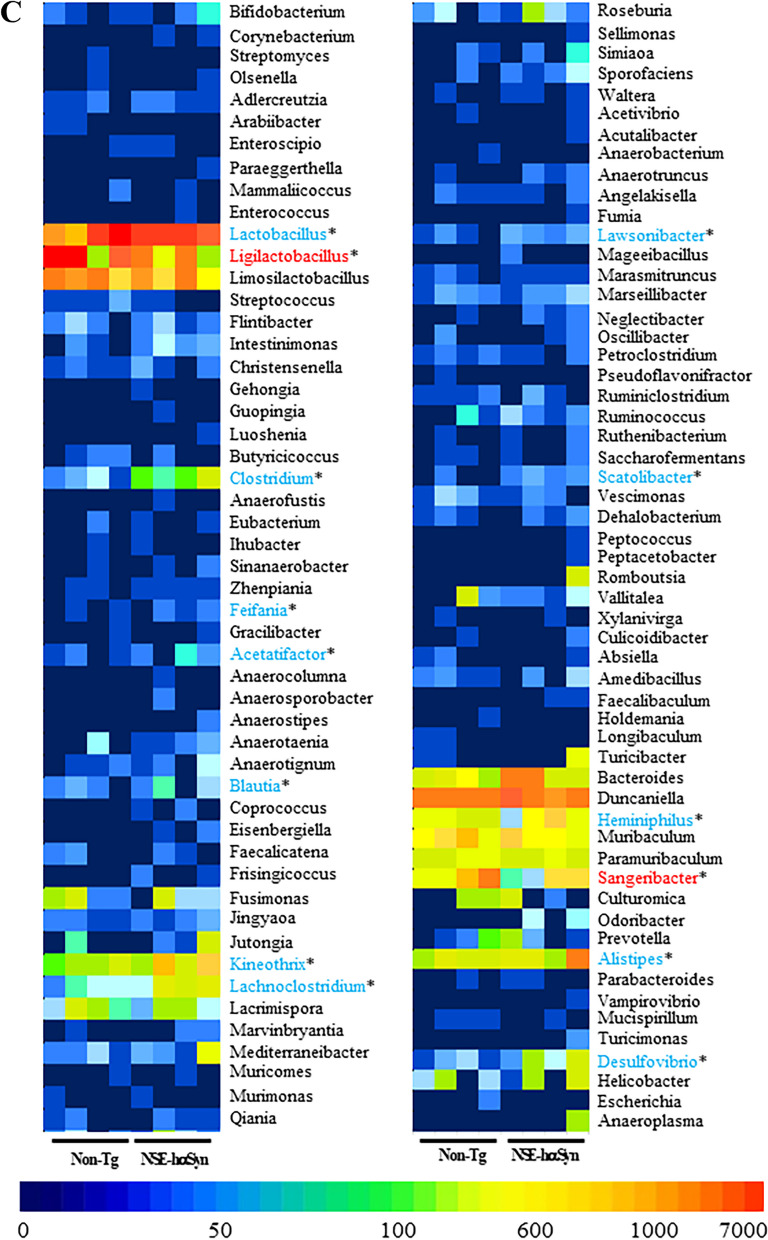
Characterization of the fecal microbiota. **A** Fecal microbiota distribution at the genus level in Non-Tg and NSE-hαSyn Tg mice. **B** Alteration on the genus with more than 1000 microorganisms. **C** Heat map showing a significant difference between Non-Tg and NSE-hαSyn Tg mice at the bacteria genus level. The data are presented as the mean ± SD. *P < 0.05 versus Non-Tg group. Abbreviation: *hαSyn* human α synuclein, *NSE* neuron-specific enolase, *Tg* transgenic mice

**Fig. 7:**
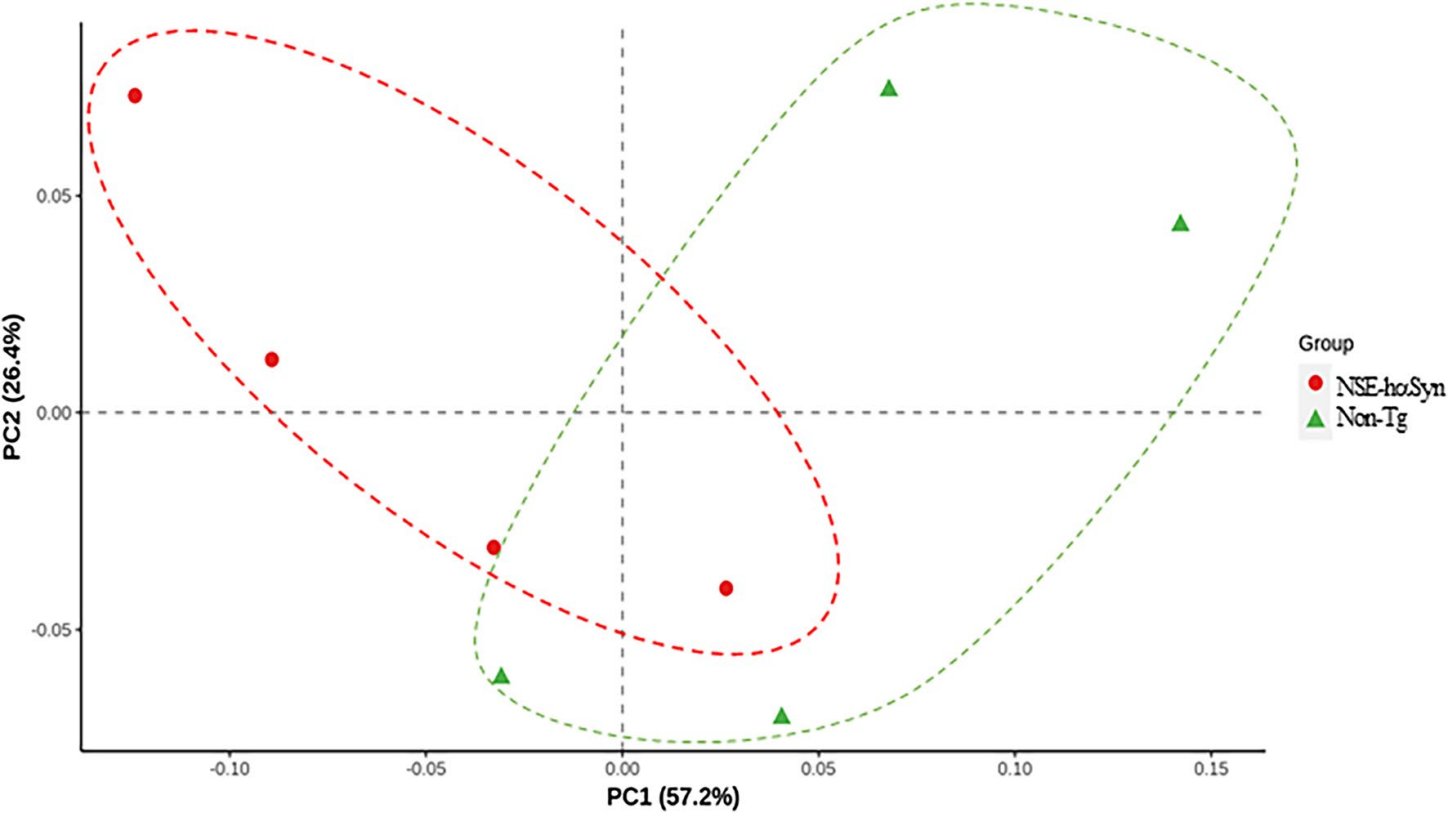
Dissimilarity analysis using a distance matrix with Bray Curtis dissimilarity of all populations between Non-Tg and NSE-hαSyn Tg mice. PCoA map can be defined based on the distance matrix between samples to observe the differences in microbial populations between samples. Abbreviation: *hαSyn* human α synuclein, *NSE* neuron specific enolase, *Tg* transgenic mice

**Fig. 8:**
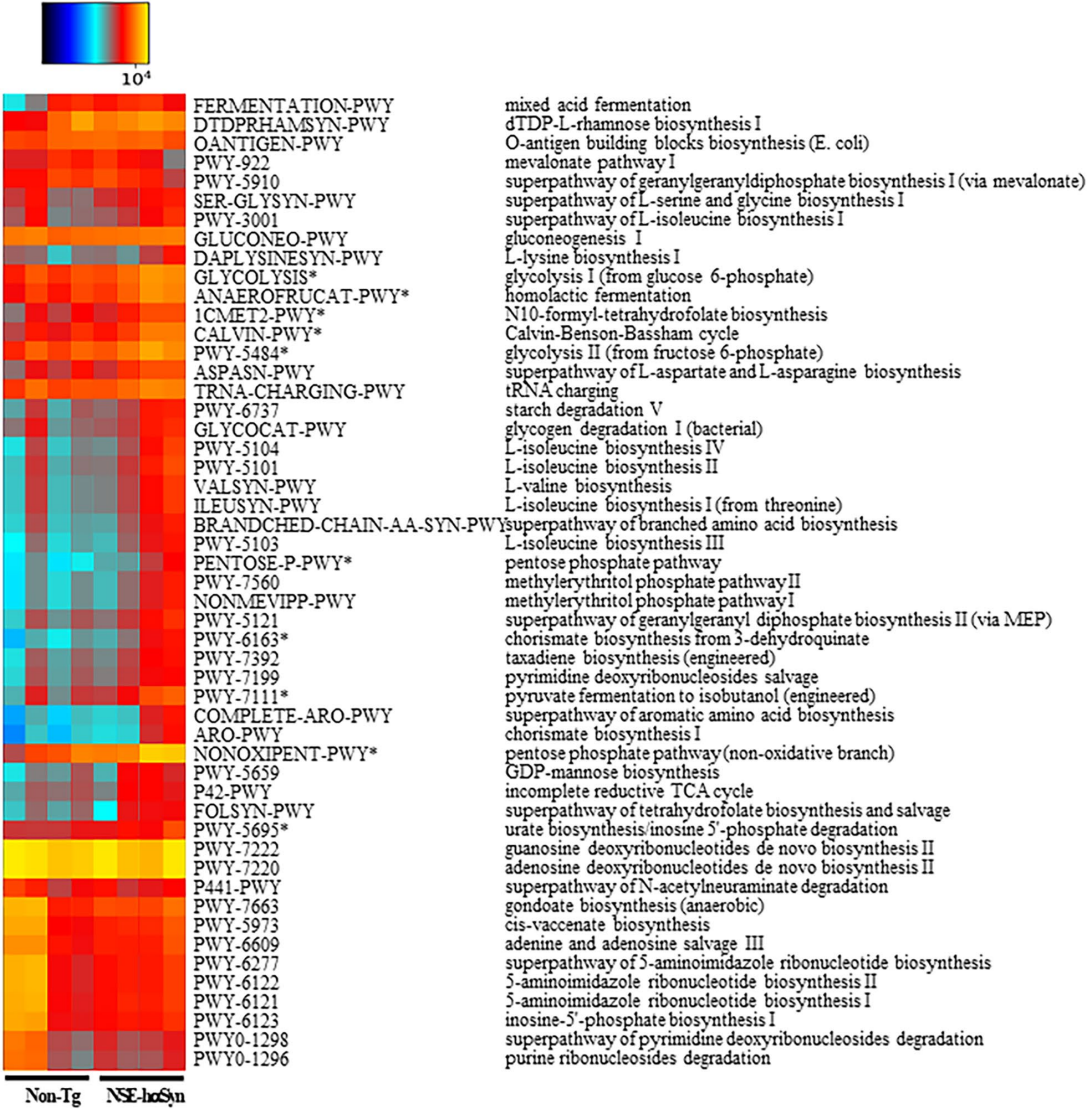

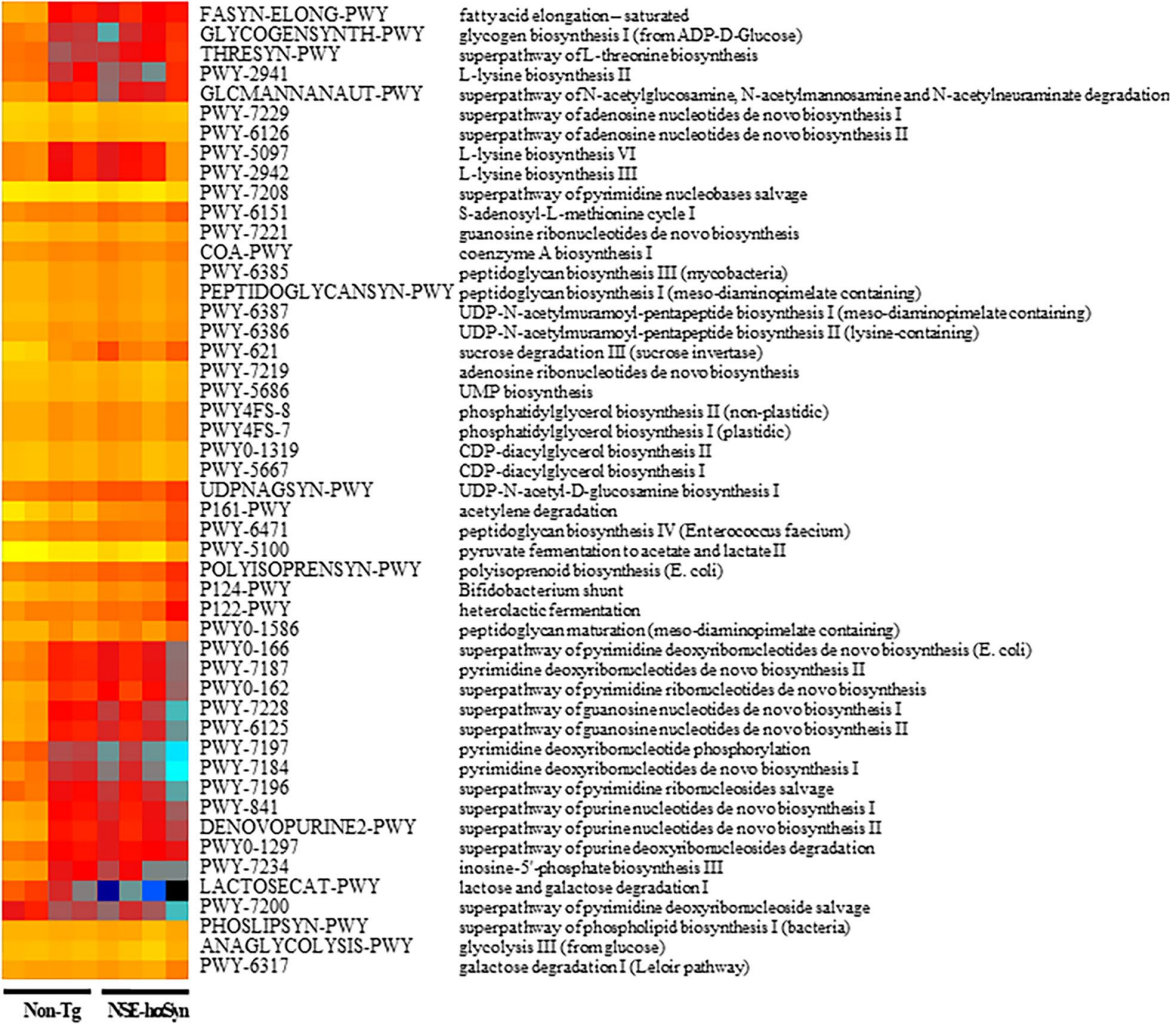
Relative abundance of the pathways predicted by PICRUSt2 in NSE-hαSyn Tg mice. Clustered heatmaps of relative abundance of the predicted pathway were differentially abundant between Non-Tg and NSE-hαSyn Tg mice. The color of the heatmap indicates the z-score normalized relative abundances. Abbreviation: *hαSyn* human α synuclein, *NSE* neuron-specific enolase, *Tg* transgenic mice

Finally, this study analyzed the overall microbial composition in fecal samples of NSE-hαSyn Tg mice to determine if the profile of the fecal microbiota is affected by hαSyn-induced PD. The Chao index and Shannon index showed that the richness of microbial species in NSE-hαSyn Tg mice was increased significantly from that of the Non-Tg mice (p = 0.035) although the increase in their diversity is not significant (p = 0.117) (Fig. 5). In addition, a significant alteration between the Non-Tg and NSE-hαSyn Tg mice was detected in 11 microbial genera. Among them, the population of only two genera, including *Ligilactobacillus* (75%) and *Sangeribacter* (53%), were remarkably decreased in the fecal sample of NSE-hαSyn Tg mice compared to the Non-Tg group. Most of them, including Scatolibacter, Clostridium (9.4 times), Clostridium (5.6 times), Feifania (5.5 times), Lachnoclostridium (5.4 times), Acetatifactor (5.1 times), Lawsonibacter (4.8 times), Desulfovibrio (4.5 times), Kineothrix (2.8 times), Blautia (2.2 times), Alistipes (2.0 times) and Heminiphilus (1.2 times) population at the genus level, were increased in the same group (p = 0.004) (Fig. 6). Furthermore, the colony structure of the fecal microbiota was compared by principal coordinate analysis (PCoA) based on the Bray–Curtis dissimilarity matrix. Cluster separation was detected between the Non-Tg and NSE-hαSyn Tg mice (Fig. 7). By PICRUSt2, 293 metabolic pathways were predicted from the data. These pathways consisted of three major groups, including biosynthesis (166, 56%), superpathway (89, 30%), and degradation (56, 19%)(p = 0.039 in Glycolysis, p = 0.036 in Anaerofrucat-Pwy, p = 0.046 1in Cmet2-Pwy, p = 0.041 in Calvin-Pwy, p = 0.04 Pwy-5484, p = 0.037 Pentose-P-Pwy, p = 0.036 in Pwy-6163, p = 0.038 in Pwy-7111, p = 0.033 in Nonoxipent-Pwy, p = 0.031 in Pwy-5695). Figure 8 shows the top 100 metabolic pathways. Therefore, the motor dysfunction of NSE-hαSyn Tg mice may be closely linked to the dysbiosis of the fecal microbiota in NSE-hαSyn Tg mice at the genus levels.

## Discussion

PD was characterized by the degeneration of dopaminergic neurons in the substantia nigra, and the formation of Lewy bodies (LB) aggregated by αSyn in other neurons [16]. In addition to these characteristics, the GBA was recently verified in PD based on an analysis of the correlation between the misfolding and deposition of α-Syn in ENS and CNS, and the imbalance of the gut microbiota [17–19]. Several microbiological techniques, including fecal microbiota transplantation (FMT), have been considered novel therapeutic strategies to improve PD symptoms [20]. This study examined the potential of NSE-hαSyn Tg mice as a GBA model through the analyses of gut microbiota and the detection of PD phenotypes. In the present study, compositional changes of gut microbiota were detected in NSE-hαSyn Tg mice with overexpression of hαSyn protein, decreasing TH and dysfunction of motor balance. The present results show that overexpression of hαSyn under the NSE promoter can induce PD symptoms and may be associated with changes in the gut microbiota composition. In addition, these results provide evidence that NSE-αSyn Tg mice can be used as a GBA model.

Several remarkable phenotypes for PD were detected in genetically engineered mice (GEM) and chemical-induced model. Among them, Thy1-αSyn Tg mice are the best-known model for PD. In these mice, hαSyn proteins accumulated in various brain regions, including the neocortex, olfactory region, limbic system, basal ganglia, and substantia nigra [21]. A severe dysfunction of motor activity was detected in beam traversal, hindlimb clasping reflexes, pole descent, and nasal adhesive test, even though studies show no difference between four and 12 weeks [10, 11, 22, 23]. In addition, the Dcf1 KO mice showed several PD-like phenotypes, including learning and memory deficits, slow movement, and anxiety [13, 24, 25]. Progressive motor dysfunction was detected in the SNCA p.A53T mice at 10 months old [26]. Furthermore, the neurotoxin model of PD induced by administering MPTP exhibits a decrease in dopamine concentration, which was reflected in damage to dopaminergic neurons [12]. In this study, other Tg mice overexpressed hαSyn protein under the control NSE promoter. The level of hαSyn protein expression was higher in the substantia nigra and striatum of NSE-hαSyn Tg mice than in Non-Tg mice. These changes were reflected in the dysfunction of motor activity. Most results from the present study for the PD phenotypes related to motor dysfunction in NSE-hαSyn Tg mice were similar to previous studies that analyzed the motor activity and accumulation of αSyn protein in the chemical-induced model and GEM. On the other hand, the results of the behavioral analyses are more diverse in previous studies than in the present study.

Meanwhile, many recent researchers have actively studied the regulation of gut microbiota in PD animal models to verify the correlation between dysbiosis of microbiota and PD phenotypes [27–29]. However, most of them were focused on the PD models induced by several toxic chemicals including MPTP, rotenone and 6-hydroxydopamine [30–32]. Only few studies have examined the changes in the gut microbiota of PD model mice induced by overexpression and deletion of specific target genes [10, 11]. Among them, significant alterations on the composition of microbiota were detected in two transgenic mice overexpressed human αSyn proteins. Thy1-αSyn Tg mice showed remarkable changes in eight microbial families, including *Proteus* sp., *Bilophila* sp., *Roseburia* sp., *Lachnospiraceae*, *Rikenellaceae*, *Peptostreptococcaceae*, *Butyricicoccus* sp., and *Verrucomicrobiae* [10, 11]. A53T missense mutation of αSyn (A53T-αSyn) overexpression mice exhibited an increased level of two microbial populations, such as *Parabateroides* and *Ruminococcus*, compared to the control, while α-ketoglutarate (AKG)-based diet significantly changed *Lachnospiraceae_NK4A136_group* in A53T-αSyn Tg mice [26, 33]. In addition, the microbiota composition in Dcf1KO mice was altered significantly in *Proteobacteria* and *Prevotellaceae* during the induction of PD-like phenotypes [13]. Based on all the above results, *Proteobacteria*, *Prevotellaceae*, and *Lachnospiraceae* can be considered the most commonly altered microorganism during PD symptoms. This study investigated whether the overexpression of hαSyn protein under the control NSE promoter can induce microbial composition alterations in the GI tract. The abundance of 11 microorganisms at the genus level was remarkably higher in the NSE-hαSyn Tg mice compared to the control, while those of two microorganisms were lower in the same group. Most of these results at the genus level differed from previous studies. These differences in the age of the analyzed mice were expected to be one of major factors affecting the number of altered microbe genera significantly although various causing factors including diet, drinking water, breeding condition and affected genes can contribute to these differences.

## Conclusions

This study examined the compositional changes in fecal microbiota of NSE-hαSyn Tg mice to evaluate the potentials as a GBA model. The changes in the microbial composition of feces and PD phenotypes were analyzed in 90-week-old NSE-hαSyn Tg mice. At the genus levels, the abundance of 13 microbial populations was changed significantly in the NSE-hαSyn Tg mice during defect of motor activity. Therefore, the NSE-hαSyn Tg mice can be considered a novel mouse model for GBA study. On the other hand, further researches using the FMT technique, antibiotic treatment or co-housing experiments are required to verify the critical role of compositional changes for microbiota in αSyn accumulation-induced PD. Moreover, the lack of scientific evidence for a clear mechanism of action on the relationship between microorganisms and PD symptoms in this model should be considered as a limitation of our study.

## Methods

### Care and use of experimental animals

The experimental protocol was approved by the Pusan National University-Institutional Animal Care and Use Committee (PNU-IACUC, Approval Number PNU-2022-0139) and Korea National Sport University-IACUC (KNSU-IACUC-2023-04) based on the ethical procedures for scientific care. All mice were housed at the PNU-Laboratory Animal Resources Center (LARC) accredited by the Korean Food and Drug Administration (KFDA) (unit 000231) and the Association for Assessment and Accreditation of Laboratory Animal Care International (AAALAC International) (unit 001525) as well as Laboratory Animal Room of Korea National Sport University. The standard irradiated chow diet (Samtako BioKorea Inc., Osan, Korea) was provided to all mice during the experimental period, and was allowed to be ingested freely with tap water (*ad libitum*). The mice were breed at the facility where the temperature of 23 ± 2°C, the relative humidity of 50 ± 10%, a strict light cycle (on at 08:00 h; off at 20:00 h) and a specific pathogen-free (SPF) state were maintained.

NSE-hαSyn Tg mice were overexpressed hαSyn protein under the control of the NSE promoter (Additional file 1: Fig. S1). The 90-week-old NSE-hαSyn Tg mice with C57BL/6Korl background (male, n = 9) and the same age of Non-Tg mice (male, n = 9) was kindly provided by the Department of Laboratory Animal Resources of the NIFDS (Chungju, Korea). After the final rotarod test, all mice were euthanized with CO_2_ gas with a minimum purity of 99.0% according to the AVMA Guidelines for the Euthanasia of Animals. A cage containing animals was placed in the chamber, and CO_2_ gas of 99.0% was introduced into the chamber without pre‑charging, with a fill rate of ~ 50% of the chamber volume per minute. The death of the mice was confirmed by cardiac and respiratory arrest or dilated pupils and fixed bodies. The brain and total gastrointestinal (GI) tract samples were collected from all euthanized Non-Tg and NSE-haSyn Tg mice.

### Rotarod test

A Rotarod test was performed to evaluate fore and hind limb motor coordination and balance impairments, as described elsewhere [34]. The mice were briefly trained in the Rotarod (Daejong Lab, Seoul, Korea) at 10–20 rpm until all mice reached a stable performance (for baseline). The preliminary test was conducted on the first day, and the main test was conducted on the second day. During the main test, each mouse was placed on a rotating tube at a steady speed of 10 rpm. The rotating speed was increased gradually from 10 to 20 rpm in 200 s, and the final speed was then maintained for at least 300 s with two more attempts. The running time was collected and expressed as the fall times (s) and number (ea) for each mouse.

### Western blotting analysis

The total proteins were collected from the brain (Substantia nigra and striatum) of Non-Tg or NSE-haSyn Tg mice using the Pro-Prep Protein Extraction Solution (Intron Biotechnology Inc., Seongnam, Korea) based on the company’s recommended manual. After collecting total brain homogenate, their concentrations were measured using a SMARTTM Bicinchoninic Acid Protein assay kit (Thermo Fisher Scientific Inc., Wilmington, MA, USA). About 30 µg of brain proteins were loaded to 4–20% Mini-PROTEAN® TGX™ Precast Protein Gels electrophoresis (SDS-PAGE) for 90 min, and the resolved proteins were transferred to a Trans-Blot Turbo Mini 0.2 µm Nitrocellulose Transfer Packs for 3 min. The membranes were then probed overnight with the following primary antibodies at 4°C: anti-αSyn antibody (Santa Cruz Biotechnology, Santa Cruz, CA, USA), anti-TH antibody (Santa Cruz Biotechnology, Santa Cruz, CA, USA), or Ponceau (Sigma–Aldrich, St. Louis, MO, USA). The resulting membranes were washed with a washing buffer (137 mM NaCl, 2.7 mM KCl, 10 mM Na_2_HPO_4_, 2 mM KH_2_PO_4_, and 0.05% Tween 20), followed by incubation with 1:1000 diluted horseradish peroxidase-conjugated goat anti-rabbit IgG (Zymed Laboratories, South San Francisco, CA, USA) for 2 h at room temperature. The blots were then developed using a Chemiluminescence Reagent Plus Kit (Pfizer Inc., Gladstone, NJ, USA). The signal images of each protein were acquired using a digital camera (1.92 MP resolution) of the FluorChem® FC2 Imaging system (Alpha Innotech Corporation, San Leandro, CA, USA). The protein densities were semi-quantified using the AlphaView Program, version 3.2.2 (Cell Biosciences Inc., Santa Clara, CA, USA).

### Histopathological analysis

After being separated by four specific areas, including duodenum, jejunum, ileum, and colon, the samples were fixed in a 10% formalin solution for 48 h at room temperature. The middle region of each tissue was embedded into a paraffin block. After sectioning the tissue block into 4 mm thick slices, these sections of the GI tract were stained with an H&E solution (Sigma–Aldrich; Merck KGaA) for 3–4 min at room temperature. Finally, the villus and crypt regions on the stained section were observed by optical microscopy (Leica Microsystems, Glattbrugg, Switzerland), and subsequentially thickness and length of them were measured using the Leica Application Suite X Microscope Software (Leica Microsystems).

### Immunohistochemical analysis for brain

Immunohistochemistry (IHC) was performed as described elsewhere [35]. Briefly, Non-Tg and NSE-hαSyn Tg mice were anesthetized using intraperitoneal injection of Alfaxan (JUROX Pty Limited, Rutherford, Australia, 13 mg/kg body weight i.p.). The mice were perfused transcardially with 1 × PBS followed by 4% formaldehyde to remove the blood and fix the brain tissue. After perfusion, the fixed brain was collected from the skull of each mouse and fixed overnight in formaldehyde, after which each brain was dehydrated and embedded in paraffin. A series of brain sections (10 μm) were cut from the paraffin-embedded tissue using a Leica microtome (Leica Microsystems, Bannockburn, IL, USA). The brain sections were deparaffinized with xylene, rehydrated, and pretreated for 30 min at room temperature with PBS-blocking buffer containing 10% normal goat serum (Vector Laboratories Inc. Burlingame, CA, USA). The samples then underwent IHC analyses. The sections were then incubated with primary mouse anti-TH antibody (Santa Cruz Biotechnology, Santa Cruz, CA, USA) for 24 h, and incubated in secondary antibody for 2 h at room temperature. The reagents that reacted with ABC (Avidin–Biotin Complex) were visualized by stable 3,3′-diaminobenzidine (DAB; Invitrogen, Carlsbad, CA, USA) as a substrate at room temperature for 2 min, counterstained with hematoxylin, dehydrated, and mounted with coverslips. Finally, the color distribution on the brain section was observed under the optical microscopy (Leica Microsystems).

### Fecal microbiota analysis

The fecal microbiota analyses were performed as described in previous study [36]. After collecting the total DNA from the fresh feces (1 g) from a single mouse (n = 4 in the subset group) using a DNeasy Power Soil Kit (Qiagen, Hilden, Germany), the sequencing libraries were prepared based on the Illumina 16S Metagenomic Sequencing Library protocols. The products of genes for 16 s rRNAs were amplified by 1st and the 2nd PCR process using Universal primer and NexteraXT Indexed primer, and purified based on the qPCR Quantification Protocol Guide (KAPA Library Quantification kits for IlluminaSequencing platforms). The sequence of these PCR products was analyzed by the paired-end (2 × 300 bp) sequencing method using the Macrogen unit on the MiSeq™ platform (Illumina, San Diego, CA, USA). Their data were curated using the Fastp program [37] and assigned to Operational Taxonomic Units (OTUs) using the Cluster Database at High Identity with Tolerance (CD-HIT-OUT) [38]. Also, sequence of each OTU was aligned according to BLAST+ (v2.9.0) [39] in the Reference DB (NCBI 16S Microbial), and analyzed using the MAFFT (v7.475) program. Furthermore, the comparative analysis of several microbial clusters was analyzed using QIIME (v1.9) [40]. The Shannon Index was used to obtain the α-diversity information, and the Rarefaction curve nd Chao1 values were used to verify their information. Moreover, the Weighted/Unweighted UniFrac distance was applied to determine the β-diversity, while PCoA was used to visualize the flexibility [40]. In addition, the PICRUSt2 (Phylogenetic Investigation of Communities by the Reconstruction of Unobserved States, Huttenhower Lab, Boston, MA, USA) was utilized to predict the MetaCyc metabolic pathway of the fecal microbiota. The ggplot (v3.3.2) program was applied to visualize the function of each microbiota, and PCoA analyses were used to represent the dissimilarity between microbiota.

### Statistical analysis

The statistical significance between Non-Tg mice and NSE-haSyn Tg mice was evaluated using the Student t-test (SPSS for Windows, Release 10.10, Standard Version, Chicago, IL, USA). All values are expressed as the means ± SD; p-values (p < 0.05) were considered significant.

### Supplementary Information

**Additional file 1. Fig. S1**: Identification of in NSE-hαSyn Tg mice. (**A**) Vector map. The hαSyn gene was constructed with SV40 Poly(a) terminator under NSE promoter. (**B**) PCR typing of tail DNA. PCR products (600 bp size) amplified from the tail of NSE-haSyn Tg mice were detected by agarose gel electrophoresis.

## Data Availability

The datasets used and analyzed during the current study are available from the corresponding author upon reasonable request.

## Abbreviations

AAALAC: Association for Assessment and Accreditation of Laboratory Animal Care
AD: Alzheimer’s disease
CNS: Central nervous system
Dcf1: Dendritic cell factor 1
dNTP: Deoxynucleotide
ENS: Enteric nervous system
FMT: Fecal microbiota transplantation
GBA: Gut–brain axis
GEM: Genetically engineered mice
GI: Gastrointestinal
hαSyn: Human α-synuclein
HPA axis: Hypothalamic–pituitary–adrenal axis
ICC: Interstitial cells of Cajal
KO: Knockout
LB: Lewy bodies
MPTP: Methyl-4-phenyl-1,2,3,6-tetrahydropyridine
NSE: Neuron specific enolase
OTUs: Operational Taxonomic Units
PCR: Polymerase chain reaction
PD: Parkinson's disease
SDS-PAGE: Sodium dodecyl sulfate–polyacrylamide gel electrophoresis
Tg: Transgenic
WT: Wild type
