# Supplementary figures and images for: Compositional changes in fecal microbiota in a new Parkinson's disease model: C57BL/6-Tg(NSE-haSyn) mice
Source: Lab Anim Res. 2023 Nov 15;39:30. doi: 10.1186/s42826-023-00181-4 (PMC10647134; doi:10.1186/s42826-023-00181-4)

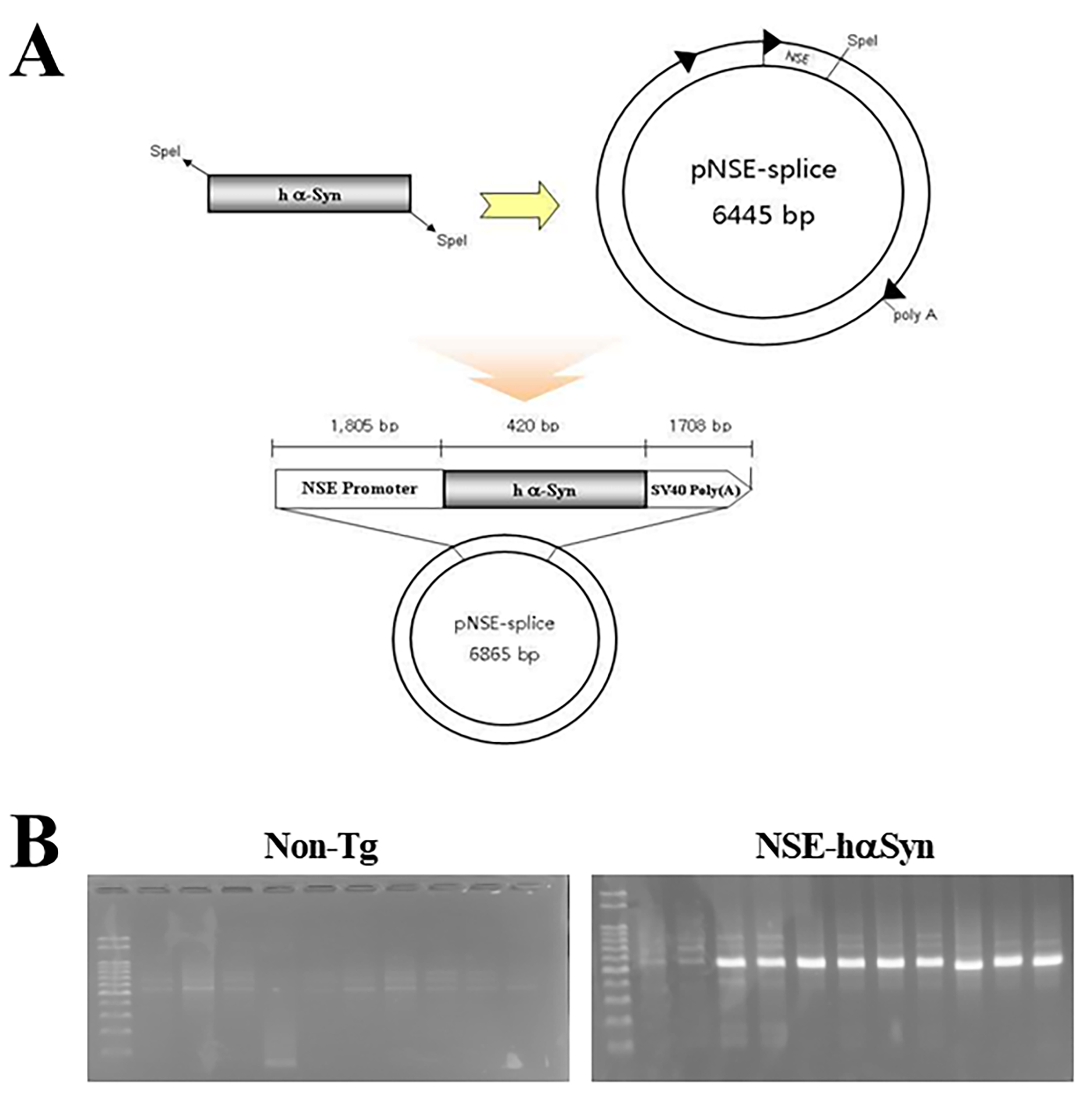

Supplement: Supplementary file 1 — Additional file 1. Fig. S1: Identification of in NSE-hαSyn Tg mice. (A) Vector map. The hαSyn gene was constructed with SV40 Poly(a) terminator under NSE promoter. (B) PCR typing of tail DNA. PCR products (600 bp size) amplified from the tail of NSE-haSyn Tg mice were detected by agarose gel electrophoresis. [file 42826_2023_181_MOESM1_ESM.tif]
